# Hydrophilic nanosilica as a new larvicidal and molluscicidal agent for controlling of major infectious diseases in Egypt

**DOI:** 10.14202/vetworld.2017.1046-1051

**Published:** 2017-09-11

**Authors:** Marwa M. Attia, Soliman M. Soliman, Mahmoud A. Khalf

**Affiliations:** 1 Department of Parasitology, Cairo University, Giza, P.O. Box 12211, Egypt; 2 Department of Medicine and Infectious Diseases, Cairo University, Giza, P.O. Box 12211, Egypt; 3 Department of Veterinary Hygiene and Management, Faculty of Veterinary Medicine, Cairo University, Giza, P.O. Box 12211, Egypt

**Keywords:** *Biomphalaria alexandrina*, *Culex pipiens*, Egypt, nanosilica, rift valley fever, schistosomiasis

## Abstract

**Aim:**

This research was conducted to evaluate the molluscicidal and mosquitocidal efficacy of silica nanoparticles in the eradication of the larvae and pupa of malaria and filariasis vector as well as vectors of rift-valley fever virus *(Culex pipiens*); *Schistosoma mansoni* vector and *Biomphlaria alexandrina* (snail and egg masses).

**Materials and Methods:**

Hydrophilic nanosilica particles (NSPs) were characterized using transmission electron microscope during the preliminary part of the study; the stages were exposed to upgrade concentrations of NSP from 50 to 1200 ppm each for 24-36 h exposure time. The highly effective concentrations were re-evaluated at lower exposure time as 3, 6, and 12 h.

**Results:**

Lethal concentration (LC_50_) and LC_90_ versus mosquito larvae were (350 ppm/24 h and 1400 ppm/24 h, respectively). *C. pipiens* pupae proved slight high tolerance versus the effect of these nanoparticles as the two previous doses increased to 680 ppm/6 h and 1300 ppm/24 h. The LC_50_ and LC_90_ versus *B. alexandrina* were increased to 590 ppm/6 h and 980 ppm/48 h, respectively. Moreover, the embryonated snail egg masses appear more susceptible to the toxic effect of these nanoparticles than the non-embryonated eggs as the LC_50_ and LC_90_ were increased to 1450 ppm/12 h and 1250 ppm/48 h, respectively, for embryonated eggs, and it was 1400 ppm/24 h and 1890 ppm/48 h, respectively, for non-embryonated one.

**Conclusion:**

The results open a new field for controlling the infectious diseases through eradication of their vectors by the way that avoids the resistance recorded from the successive chemical application in this field.

## Introduction

Culex pipiens is the common house mosquito. It serves as a vector of several diseases, including St. Louis encephalitis virus, West Nile virus, and rift valley fever. It causes insect worry and filariasis by Wuchereria bancrofti in humans [1]; also it transmits Dirofilaria immitis and Dirofilaria repens (dirofilariasis) to dogs [2].

Schistosomiasis is a widespread parasitic disease affecting more than 200 million people worldwide [3,4]. It is an important disease in Egypt; the disease is transmitted through widely spreading intermediate host *Biomphalaria alexandrina* [5]. One of the control studies to combat schistosomiasis is to destroy the vector life cycle in endemic areas through control of the snail’s population [6].

A few years ago; uses of synthetic insecticides in modern agriculture production, promote the wide spread of environmental contamination, toxicity to human food as well as resistance was developed and hazardous to human health [7]. Hence, controlling of insect required a modern technology of fewer hazards to human health with no residues in their food and less resistance to organisms.

Nanomedicine is a new promising field study for scientists in veterinary technology which deals with production and use of materials ranging in nanometers of different size and shapes. Research and development are now focusing on applications of these nanomaterials on human health (drug delivery, cancer therapeutics, catalysis, and larvicidal against parasite); this leads to a production of the new biocidal agent against some parasites [8].

Nanosilica was reported to have a potential as a drug delivery for medical and veterinary diseases as pesticides. International Agency for Research on Cancer [9] decided that amorphous silica is not regarded as carcinogenic materials unlike crystalline silica [10].

The present study is a trial to characterize the selected hydrophilic nanosilica particles (NSPs) using transmission electron microscope (TEM) followed by an evaluation to the molluscicidal and mosquitocidal efficacy of these particles versus *B. alexandrina* snail and their egg masses as well as versus larvae and pupa of *C. pipiens* under controlled laboratory conditions.

## Materials and Methods

### Ethical approval

This research was approved by the Scientific Research Ethical Committee, Faculty of Veterinary Medicine, Cairo University.

### Silica nanoparticle

Silica nanoparticles 99% purity were purchased from Nanotech (Egypt); characterized using TEM; imaging was performed using a Jeol-JEM Japan 2100 operating at 80 KV. The sample was sonicated in ethanol and deposited onto copper coated carbon grid and left to evaporate. Finally, the specimens were examined and photographed [11] at Faculty of Agriculture, Cairo University, Egypt.

### Mosquito rearing

Eggs of *C. pipiens* were obtained from the Medical Research Institute of Insects, Giza, Egypt. The eggs were floated on dechlorinated tap water in suitable plastic aquaria (40 cm × 60 cm × 10 cm) at room temperature (26±1°C) to developed into the first larval stage [12]. Larvae were reared in the same aquarium, and the required number and stage of development (larvae-pupa) were selected and exposed separately to the tested chemicals.

### Tested snails

*B. alexandrina* snails were collected from irrigation canals at Abu Rawash locality, Giza governorate. The snails were identified by Chrsistensen *et al*. [13]. They were maintained in the laboratory conditions at room temperature (26±1°C) in dechlorinated tap water, fed with fresh lettuce leaves with daily cleaning for at least 7 days before being used in the experiment. The aquaria were supplied with clean cellophane sheet for collection of the deposited snail egg masses. Snails were screened for natural infection with trematode larvae [14]. Medium size trematode free active snails were separated and being used for screening the molluscicidal activity of nanosilica (NSP). Moreover, early deposited and pre-hatched intact embryonated snail egg mass on cellophane sheet were selected for exposure.

### Bioassay test

The larvicidal activity was assessed by the WHO [15] with little modification. Molluscicidal activity was evaluated according to the WHO immersion technique [1] with slight modifications. The experiments were carried out at a room temperature at 26±2°C and pH 7.4 with a photoperiod of 14 h/10 h (light/dark). Efficacy of the tested NSP was evaluated on *C*. *pipiens* (early 2^nd^ larvae, pupa) and *B*. *alexandrina* (snails, non-embryonated, and embryonated egg masses). Each stage was exposed separately in four replicates (25 agents each) in 100 ml glass jar. A preliminary evaluation of the effect of NSP has applied on *B. alexandrina* and *C. pipiens* larva as they were exposed to upgraded concentrations of NSP as 50, 100, 200, 400, 600, 800, 1000, and 1200 ppm, each for 24, 48, and 36 h exposure time in dechlorinated tap water. Concentrations which were induced as 50% mortalities or above were rescreened at the lower exposure times (3, 6, 12, 24, and 48 h) to determine the minimum exposure periods causing considerable mortalities in the exposed stages. Lethal concentration (LC_50_) and LC_90_ were determined from all the previous trailers. LC_100_ of copper sulfate (30 ppm) and that of temephos (0.04 mg/L) were used as reference molluscicides and mosquitocides [14,16]. Their concentration and exposure time were obtained according to the manufacture guidelines.

### Evaluation of mortalities

At the end of each exposure period, the chemicals were removed, and the stages were washed several times by dechlorinated tap water, transferred to clean beakers and kept under observation for the evaluation of mortality percentage 24 h post-exposure [17]. Mortality in snails was checked using the crushing technique (5% sodium hydroxide solution) [1], snails were considered dead if they remained motionless and did not respond to the presence of food. The efficacy was estimated from the mean mortality of the four replicates [10]. The designed curve between mortality % in relation to the tested silver nanoparticle concentrations and exposure time was used to calculate the LC_50_ and LC_90_ according to probit analysis [18,19]. The experiments were conducted simultaneously; control larval, pupa, and snails tested in dechlorinated tap water and run with each experiment.

### Statistical analysis

Data were analyzed with SPSS version 21, with confidence intervals 95% determined by probit analysis [19]. LC_50_ and LC_90_ values were calculated through drawing a transverse line pass from probit 0.5 in the y-axis, then move down to the x-axis and find the log concentration.

## Results

**Figure-1:**
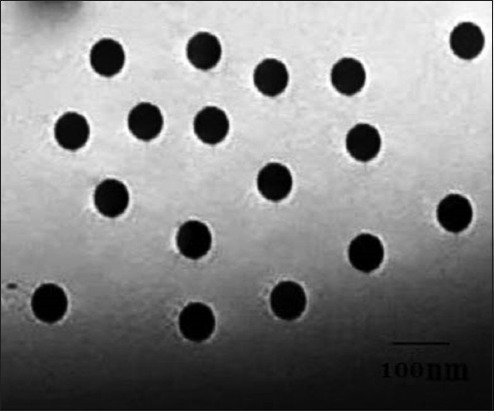
Transmission electron microscope of nanosilica showing its rounded shape with its diameter.

TEM image of the used silica nanoparticles (Figure-1) proved that they are spherical in shape with the size range of 80 nm in diameter. Exposure of *C. pipiens* larvae and *B. alexandrina* snails to different concentrations of the tested hydrophilic NSP revealed their ability to induce mortalities in the exposed stages in comparison with the control stages in dechlorinated tap water. There is a direct relationship between the mortality of the mosquito and snail with increasing concentration and the exposure time for NSP solution. Concerning the preliminary part of the study (Table-1), the concentrations <50 ppm did not cause mortalities in the exposed stages. Mortalities in *C. pipiens* larvae start as 21±0.3% after exposure to 50 ppm/24 h. This effect increases with time versus the same concentration as it reached to 32±0.36% after 36 h exposure. With increasing of the particle concentration to 200 and 400 ppm, their mosquitocidal effects increased from 48±0.4% and 68±0.43% after 24 h to 66±0.43% and 87±0.37% after 36 h exposure for both concentrations, respectively. After increasing the concentration to 1000 and 1200 ppm this effect increased to 80±0.27% and 82±0.34% after 24 h exposure then reached to 100% after 36 h exposure time (Table-1).

**Table-1:** Molluscicidal and mosquitocidal efficacy of NSP (preliminary screening).

Tested concentration (ppm)	*C. pipiens* 2^nd^ stage larvae			*B. alexandrina* snails		
	
	MM%±SE after exposure			MM%±SE after exposure		
	
	24 h	48 h	36 h	24 h	48 h	36 h
50	21±0.3	28±0.21	32±0.36	18±0.21	22±0.21	28±0.34
100	28±0.25	36±0.24	41±0.34	24±0.32	32±0.20	38±0.48
200	48±0.4	61±0.43	66±0.43	38±0.35	58±0.34	61±0.31
400	68±0.43	81±0.45	87±0.37	63±0.37	78±0.46	82±0.40
600	71±0.53	85±0.37	89±0.48	69±0.43	82±0.44	86±0.42
800	77±0.62	91±0.46	96±0.43	71±0.34	88±0.32	93±0.48
1000	80±0.27	94±0.43	100±0.00	76±0.49	93±0.45	100±0.00
1200	82±0.34	100±0.00	100±0.00	79±0.38	100±0.00	100±0.00
Sig.	0	0.001	0	0.001	0.001	0.003

No mortalities in control groups in dechlorinated tap water during the same exposure periods. MM%=Mean mortality %, SE=standard error, NSP=Nanosilica particles, *C. pipiens=Culex pipiens, B. alexandrina=Biomphalaria alexandrina*

The tested NSP proved lower molluscicidal effect versus *B. alexandrina* snails than that previously recorded versus mosquito larvae. The molluscicidal effect appears as 18±0.21% after 24 h exposure increased to 28±0.34% with increasing the exposure time to 36 h versus 50 ppm NSP concentration. Increasing the concentration to 200 and 400 ppm reflects an increase in the mortalities of the exposed snails from 38±0.35% and 63±0.37% after 24 h to 61±0.31% and 82±0.40% after 36 h exposure for both concentrations, respectively. Increasing the concentration to 1000 and 1200 ppm cause increasing in their detritus effect on the exposed snails to 76±0.49% and 79±0.38% after 24 h exposure then reached to 100% after 36 h exposure time (Table-1).

Concerning the effect of NSP versus *C. pipiens* larvae and pupae, the data (Table-2) revealed that pupae appear less sensitive than larvae versus these particles. Exposures to high dose (1200 ppm) for 3 h induce low mortalities reached to 32±0.34% in larvae and 18±0.35% in the exposed pupa. Mortality percentages reached to 55±0.48% and 51±0.41% after exposure to 600 ppm for 6 h in larva and pupae, respectively. This effect increased to 59±0.39% and 55±0.33% after increasing the exposure time to 12 h for both stages, respectively. The maximum mortalities recorded in the exposed pupae 91±0.52% after exposure to 1200 ppm for 48 h (Table-2).

**Table-2:** Larvicidal and pupicidal efficacy of lethal NSP concentrations.

Tested concentration (ppm)	Exposed stages	MM% after exposure				

		3 hrs	6 hrs	12 hrs	24 hrs	48 hrs
400	Larvae	0.00	48±0.37**	52±0.34	68±0.43*	81±0.45*
	Pupae	0.00	42±0.34**	48±0.47	61±0.0*	72±0.25
600	Larvae	10±0.24	55±0.48**	59±0.39	71±0.53*	85±0.37*
	Pupae	0.00	51±0.41**	55±0.33	68±0.0*	74±0.40
800	Larvae	12±0.33	63±0.33**	68±0.42	77±0.62	91±0.46*
	Pupae	3±0.37	60±0.23**	62±0.31	70±0.0*	80±0.35*
1000	Larvae	22±0.31	72±0.34**	75±0.30	80±0.27	94±0.43*
	Pupae	14±0.24	69±0.45**	73±0.44	77±0.35	86±0.36*
1200	Larvae	32±0.34	73±0.31**	77±0.31	82±0.34	100±0.00**
	Pupae	18±0.35*	70±0.32	74±0.30	80±0.39	91±0.52*

* p<0.05,

** p>0.001,

No mortalities in control groups in dechlorinated tap water during the same exposure periods. MM%=Mean mortality %, SE=Standard error, NSP=Nanosilica particles

Nanosilica concentrations which proved considerable molluscicidal effects versus the exposed *B. alexandrina* snails during the preliminary part of the study (Table-1) were re-evaluated versus non-embryonated and embryonated *B. alexandrina* egg mass as well as the snails using lower exposure time (Table-3). NSPs at 1200 ppm concentration failed to cause mortalities on freshly deposited egg masses during exposure of 3 h, while the first effect on egg embryo was recorded using 1000 ppm for 6 h and these conditions killed only 10±0.25% of the exposed embryos. Increasing the exposure time to 24 h revealed that *B. alexandrina* snail was affected (69±0.43%) followed by pre-hatched embryonated egg masses (45±0.43%), while mortalities increase in fresh embryonated egg masses as (85±0.32%) with increasing the NSP concentrations to 1200 ppm (Table-3).

**Table-3:** Molluscicidal efficacy of NSP under different exposure time.

Tested concentration (ppm)	Exposed stages	MM% after exposure				

		3 h	6 h	12 h	24 h	48 h
600	Snail	00	52±0.37*	53±0.35	69±0.43*	82±0.44*
	FNESE	00	00	15±0.24**	25±0.31*	45±0.43**
	ESE	00	00	25±0.32**	40±0.4**	60±0.36**
800	Snail	8±0.27	60±0.38**	64±0.31	71±0.34	88±0.32*
	FNESE	00	00	15±0.30*	25±0.30*	50±0.27**
	ESE	10±0.24	25±0.32*	25±0.37	46±0.32*	62±0.36*
1000	Snail	12±0.32	65±0.42**	68±0.32	76±0.49	93±0.45*
	FNESE	00	10±0.25*	20±0.27*	40±0.43**	50±0.34*
	ESE	18±0.34	25±0.29*	35±0.28*	50±0.38**	75±0.25**
1200	Snail	18±0.28	70±0.42**	73±0.39	79±0.38	100±0.00*
	FNESE	00	10±0.25*	20±0.28*	40±0.27**	50±0.23*
	ESE	25±0.36	25±0.36	35±0.21*	60±0.38**	85±0.32**

* p≤0.05,

** p≤0.001, The results were expressed as mean±standard error.

No mortalities in corresponding groups in dechlorinated tap water during the same exposure periods. MM%=Mean mortality %, SE=Standard error, FNESE=Fresh nonembryonated snail’s eggs. ESE=Embryonated snail’s eggs, NSP=Nanosilica particles

**Figure-2:**
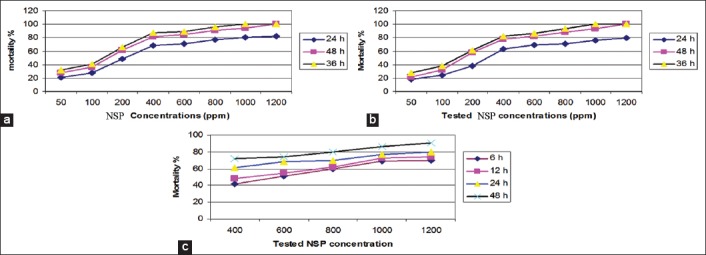
a. Relation between NSP concentration, exposure time and mortality % in *C. pipiens* larvae; b. Relation between NSP concentration, exposure time and mortality % in *B. alexandrina* snails; c. Relation between NSP concentration, exposure time and mortality % in *C. pipiens* pupa.

Using the mortality curve (Figure-2), the LC_50_ and LC_90_ for different exposed stages could be calculated as described in Table-4. LC_50_ for *C. pipiens* larvae and pupa was 350 ppm/24 h and 680 ppm/6 h, while their LC_90_ was 1400 ppm and 1300 ppm/24 h, respectively. LC_50_ for the exposed *B. alexandrina* snails, their non-embryonated egg masses and embryonated pre-hatched one were 590 ppm/6 h, 1400 ppm/24 h, and 1450 ppm/12 h, respectively. Their LC_90_ were 980, 1890, and 1250 ppm after exposure to 48 h, respectively (Table-4 and Figure-2).

**Table-4:** Mosquitocidal and molluscicidal calculated LC_50_ and LC_90_.

Exposed stages	LC_50_ (ppm)	LC_90_ (ppm)	LC_100_ (ppm)
*C. pipiens* larvae	350/24 h 280/48 h 200/36 h	1400/24 h 770/48 h 500/36 h	1200/48 h 1000/36 h
*C. pipiens* pupae	680/6 h 580/12 h	1300/24 h 980/48 h	1200/48 h
*B. alexandrina* snails	590/6 h 570/12 h 479/24 h 370/48 h	980/48 h	1200/48 h 1000/36 h
Snail fresh eggs	1400/24 h 800/48 h	1890/48 h	2000/48 h
Snails embryonated eggs	1450/12 h 1000/24 h 480/48 h	1250/48 h	1340/48 h
Copper sulfate	15 ppm		30 ppm
Temephos	0.02 mg/L		0.04 mg/L

*C. pipiens=Culex pipiens*, *B. alexandrina=Biomphalaria alexandrina*, LC=Lethal concentration

## Discussion

Among the most promising advances in the field of drug development is discovering new molecules or novel uses of the already available compounds with known safety and with minimum side effects. Development of molluscicidal, insecticidal and pesticidal substances of botanical origin may serve as suitable alternatives to synthetic ones [10,20].

Nanosilica is one of the most spreading materials on earth; it possesses a highly adhesive property to a cell membrane so can affect membrane structures. Furthermore, this nanosilica can be absorbed by phospholipid present in cuticle of the larval instar by physisorption and lysis so cause death to insects [21,22].

In this study, hydrophilic NSP was selected and identified by TEM to appear spherical in shape with a size range of 80 nm in diameter. This size proved that they are able to adhere and absorb by the surface of target agents inducing mortality [22,23].

The tested NSP revealed that they had mosquitocidal and molluscicidal efficacy starting from 50 ppm. There is a direct relation between the severity of these effects with the increase in concentration and the exposure time for these NSP solutions. Increase in the efficacy with increasing the concentrations and exposure time [10,22] with increasing the particle concentration to 200 and 400 ppm; their mosquitocidal effects increased from 48±0.4% and 68±0.43% after 24 h to 66±0.43% and 87±0.37% after 36 h exposure for both concentrations, respectively. After increasing the concentration to 1000 and 1200 ppm this effect increased to 80±0.27% and 82±0.34% after 24 h exposure then reached to 100% after 36 h exposure time.

Concerning the molluscicidal effect of the tested NSP, the effects increase also with increasing the concentration and exposure time as 200 and 400 ppm, reflects an increase in the mortalities of the exposed snails from 38±0.35% and 68±0.37% after 24 h to 61±0.31% and 82±0.40% after 36 h exposure for both concentrations, respectively.

The ability of the tested NSP to cause mortalities in both larvae, pupae, and snails may be related to this suggested mode of actions [10,22] as NSP can be absorbed by phospholipid found in cuticle of larval instar by physisorption and lysis so cause death to larval insects [10,22]. For this reason; their effect was high as the exposed tegument is thin. Hence, this study proved that the mortalities were high in larvae than in pupa. In our opinion, decreasing the mortalities in the exposed snails than larvae in the same exposure conditions may be related to the musculature nature of the snail foot, moreover *B. alexandrina* has narrow aperture, and it is able to withdraw and contract its foot inside its shell, this minimize the exposed surface to the chemical materials and decreasing mortalities. This opinion agreed to Fahmy *et al*. [18].

NSP act as larvicidal and pupicidal as its LC_50_ and LC_90_ were 350/24 h and 680/6 h and 1400, 1300/24 h, while it acts as molluscicides as its LC_50_ 590/6 h, 1400/24 h, and 1450/12 h, respectively. Their LC_90_ was 980, 1890, and 1250/48, respectively. This contrasted with Barik [10] who recorded LC_50_ (269 ppm) and LC_90_ (2006 ppm) values of hydrophilic nanosilica in *Culex quinquefasciatus*. While the efficacy of synthesized NSPs against 3^rd^ and 4^th^ instar larvae of *Aedes aegypti* and *Anopheles stephensi* were discussed and it found that LC_50_ and LC_90_ values were 1.48 and 1.58 and 3.33 and 3.41 ppm, respectively against *A. aegypti* and 1.30 and 1.41 and 3.13 and 3.29 ppm against *A. stephensi* [24].

Concerning the effect of NSP on *B. alexandrina* egg masses, the present study revealed that LC_50_ was 590/6 h versus non-embryonated eggs and it was 1400/24 h versus embryonated pre-hatched one. Moreover, LC_90_ was 980/48 h and was 1890/48 h for both stages, respectively. In the author’s opinion, this variation in the molluscicidal effect was related to the nature of structure materials of the egg masses ootheca. The ootheca is hyaline material able to protect the embryos during the period of development [18] with prolonged immersion in water, and due to movement of the developed snail embryo inside, it was liquefied and became thinner that facilitate the escape of the embryo from the ootheca. The high mortalities in embryonated eggs after exposure to NSP than that recorded in fresh non-embryonated eggs was related to the changes in the nature of ootheca with time. While Fahmy *et al*. [18] found the sub-lethal dose of zinc oxide nanoparticles against *B. alexandrina* LC_50_ and LC_90_ as 145 and 2700 µg/ml. It was reported that zinc oxide NPs showed molluscicidal activity against this snail induce malondialdehyde and nitric oxide with decreasing of glutathione and glutathione S-transferase levels in hemolymph and soft tissues so the death of this snail [18].

## Conclusion

The present study demonstrated that hydrophilic NSP can used as effective mosquitocides versus *C. pipiens* larvae when applied for 24 h at concentrations ranged between 350 and 1400 ppm or at concentration ranged between 200 and 500 ppm when applied for 36 h exposure time, so, controlling of the serious viral diseases that could be transmitted by such vectors. Moreover, it can act as effective molluscicidal versus *B. alexandrina* and their egg masses at a dose of 590 ppm after exposure to 6 h. Successful using of NSP in control of aquatic stages of vector-borne parasitic disease open new field for combating these dangerous parasites and avoided the resistance recorded from successive chemical application in this field.

## Authors’ Contributions

MMA and SMS designed the study. MMA, SSM, and MAK were involved in collection and compilation of data. MMA prepared the nanosilica for further applications. MMA, SSM, and MAK were involved in the snails’ collection as well as the bioassay testing of nanosilica for its larvicidal activity. SSM and MAK conducted the statistical analysis of the obtained results for evaluation the effect of nanosilica as a new larvicidal and molluscicidal agent for controlling of major infectious diseases in Egypt. Finally, all authors contributed to the manuscript writing and the reviewing of the literature. All authors read and approved the final manuscript.

## References

[1] WHO (2005). Guidelines for Laboratory and Field Testing of Mosquito Larvicides WHO/CDS/WHOPES/GCDPP/2005.13.

[2] Azari-Hamidian S, Yaghoobi-Ershadi M.R, Javadian E, Abai M.R, Mobedi I, Linton Y.M, Harbach R.E (2009). Distribution and ecology of mosquitoes in a focus of dirofilariasis in Northwestern Iran, with the first finding of filarial larvae in naturally infected local mosquitoes. Med. Vet. Entomol.

[3] King C.H, Dangerfield-Cha M (2008). The unacknowledged impact of chronic schistosomiasis. Chrsonic Illn.

[4] DeJong R.J, Morgan J.A, Paraense W.L, Pointier J.P, Amarista M, Ayeh-Kumi P.F, Babiker A, Barbosa C.S, Brémond P, Pedro Canese A, de Souza C.P, Dominguez C, File S, Gutierrez A, Incani R.N, Kawano T, Kazibwe F, Kpikpi J, Lwambo N.J, Mimpfoundi R, Njiokou F, Noël Poda J, Sene M, Velásquez L.E, Yong M, Adema C.M, Hofkin B.V, Mkoji G.M, Loker E.S (2001). Evolutionary relationships and biogeography of (Gasteropoda: Planorbidae) with implications regarding its role as host of the human blood fluke *Schistosoma mansoni*. Mol. Biol. Evol.

[5] Davis A, Cook G.C (1996). Schistosomiasis. Manson's Tropical Diseases.

[6] WHO (1985). The Control of Schistosomiasis. Report of a WHO Expert Committee. Technical Report Series No. 728.

[7] Pretty J, Angus C, Bain M, Barton J, Gladwell V, Hine R, Pilgrim S, Cock S.S, Sellens M (2009). Nature, Childhood, Health and Life Pathways, Interdisciplinary Centre for Environment and Society Occasional Paper 2009-02.

[8] Jones N, Ray B, Ranjit K.T, Manna A.C (2008). Antibacterial activity of ZnO nanoparticle suspensions on a broad spectrum of microorganisms. FEMS Microbiol. Lett.

[9] IARC (2007). International Agency for Research on Cancer (IARC). Summaries and Evaluations-SILICA. 2 June;2007.

[10] Barik T.K, Kamaraju R, Gowswami R (2012). Silica nanoparticle: A potential new insecticide for mosquito vector control. Parasitol. Res.

[11] Marimuthu S, Rahuman A.A, Rajakumar G, Kumar T.S, Kirthi A.V, Jayaseelan C, Bagavan A, Zahir A.A, Elango G, Kamaraj C (2011). Evaluation of green synthesized green silver nanoparticles against parasites. Parasitol. Res.

[12] Kamaraj C, Bagavan A, Elango G, Zahir A.A, Rajakumar G, Marimuthu S (2011). Larvicidal activity of medicinal plant extracts against *Anopheles subpictus* and *Culex tritaeniorhynchus*. Indian J. Med. Res.

[13] Chrsistensen N.O, Mutani A, Frandsen F (1983). A review of the biology and transmission ecology of African bovine species of the genus *Schistosoma*. Z. Parasitenkd.

[14] Chrsistensen N.O, Frandsen A (1985). An introduction to the taxonomy, morphology, biology and transmission ecology of species of the genus *Schistosoma* causing human African schistosomiasis.

[15] WHO (1996). Report of the WHO Informal Consultation on the Evaluation on the Testing of Insecticides CTD/WHO PES/IC/96.1:69.

[16] Rawi S.M, Al-Hazmi M, Al-Nassr F.S (2011). Comparative study of the molluscicidal activity of some plant extracts on the snail vector of *Schistosoma mansoni Biomphalaria alexandrina*. Int. J. Zool. Res.

[17] Lima J.B, Da-Cunha M.P, Da Silva R. C, Galardo A.K, Soares Sda S, Braga I.A, Ramos R.P, Valle D (2003). Resistance of *Aedes aegypti* to organophosphates in several municipalities in the state of Rio de Janeiro and Espírito Santo, Brazil. Am. J. Trop. Med. Hyg.

[18] Fahmy S.R, Abdel-Ghaffar F, Bakry F.A, Sayed D.A (2014). Ecotoxicological effect of sublethal exposure to zinc oxide nanoparticles on freshwater snail *Biomphalaria alexandrina*. Arch. Environ. Contam. Toxicol.

[19] Finney D.J (1971). Probit Analysis.

[20] Chuiko A.A (2003). Medical Chemistry and Clinical Application of Silicon Dioxide.

[21] Tiwari D.K, Behari J (2009). Biocidal nature of treatment of Ag-nanoparticle and ultrasonic irradiation in *Escherichia coli* dh5. Adv. Biol. Res.

[22] Barik T.K, Sahu B, Swain V (2008). Nanosilica from medicine to pest control. Parasitol. Res.

[23] Salunkhe R.B, Patil S.V, Patil C.D, Salunke B.K (2011). Larvicidal potential of silver nanoparticles synthesized using fungus *Cochliobolus lunatus* against *Aedes aegypti*(Linnaeus, 1762) and *Anopheles stephensi* Liston (*Diptera*;*Culicidae*). Parasitol. Res.

[24] Patil C.D, Patil S.V, Borase H.P, Salunke B.K, Salunkhe R.B (2012). Larvicidal activity of silver nanoparticles synthesized using *Plumeria rubra* plant latex against *Aedes aegypti* and *Anopheles stephensi*. Parasitol. Res.

